# Live, Laugh and Love—There’ll Come a Time When You Can’t

**Published:** 1911-11

**Authors:** 


### Live, Laugh and Love—There'll Come a Time When You Can't.

Here's to now—of it let us make the most! It may be that we live forever—that the soul survives, and that love and hope and happiness are the stars which Deity has set in silken skies as illimitable pledge of the riddle of existence. The lesson of this life is that nothing really worth while is wasted—that the atom is the infinite and death at most a dream—that everything was, is and will be again and again. And if all else is eternal, why not you and I? But out of the solitudes of the past and shadowy hopes and fears for the morrow glows the rosy morn of the moment—the joys of this day and hour, against which neither mummery nor mystery can prevail. Mutual recognition of soul—kinship of heart and mind—are truths as many well understand and continued association attests—who knows if, in some other age, they did not walk then as now, hand in hand? But though we be something from somewhere—though we may have lived before and may pass this way again in some far future, yet it is to the present we owe our all. Error and failure and grievous sorrow are sodded mounds o'er which time has said the service of kindly compassion. Ambition may dream of elysian fields strewn with the roses of success, and hope hath all things in store for hallowed memory and stout heart—each pointing the way through the mists of tomorrow; but it is to today after all we owe our homage—today, with its always prodigious wealth of timely fate—its golden store of opportunity.

Then here's to life and laughter and love, that trinity which amongst real men and women ne'er yet failed to wake the sphynx of the soul. Here's to blessings neither past nor promised, but present—to obligations of love and friendship not pledged, but paid—to joys not protested, but proven—to all the beauty and grace of a wondrous world which eternity has crowned with a diadem of today. What avails regret and tears—why bespeak atonement? Yesterday was—of tomorrow we know nothing. Here's to now—of it let us make the most!—*Gaillard's Southern Medicine.*

